# Shiver Me Tinders and Ring a Ding for a Fling—Sex Tech Use during COVID-19: Findings from a UK Study

**DOI:** 10.3390/healthcare11060897

**Published:** 2023-03-20

**Authors:** Hannah R. Marston, Deborah J. Morgan, Sarah Earle, Robin A. Hadley

**Affiliations:** 1 School of Health, Wellbeing and Social Care, The Open University, Milton Keynes MK7 6AA, UK; 2 Centre for Innovative Ageing, Swansea University, Swansea SA2 8PP, UK

**Keywords:** technology, gerontology, Generation X, gender, pandemic, health, dating apps, loneliness, social isolation, older adults

## Abstract

Existing research surrounding dating apps has primarily focused on younger people with few studies exploring usage of such apps by middle aged and older adults. The worldwide COVID-19 pandemic challenged social behaviours and forced people to adapt intimacy and wider relationship conduct. The objective of this study was to examine how older adults utilized dating apps during the lockdowns of the UK pandemic (December 2020–May 2021). Findings presented here focus on qualitative data collected from an online survey and eight online, one-to-one interviews with adults aged 40–54 years. The online survey targeted adults across the UK while interviewees were located across England. Employing interpretative phenomenological analysis, findings identified three key themes: 1. Morality, health, and law breaking and COVID-19; 2. Self-surveillance and moral signalling; 3. Loneliness and social isolation. Qualitative findings show engaging with apps was a proxy which alleviated feelings of loneliness and social isolation. Some users used the premise of their social bubble as a way of meeting other people. Using the same premise, others justified breaking the law to engage in physical and sexual intimacy to mitigate their loneliness. The work presented here contributes to the fields of social sciences, gerontology, and human computer interaction. The inter- and multi-disciplinary impact of this study intersects across those fields and offers a cross-sectional insight into behaviours and engagement with technology during one of the most extraordinary global events.

## 1. Introduction

The 2020 COVID-19 pandemic brought society to a standstill on a global scale [1]. Industries and routines within the United Kingdom (UK) had to adapt by adopting an agile approach to respective (devolved) government (England, Northern Ireland, Scotland, and Wales) lockdown-related directives which were deployed [2] for public safety.

The use and accessibility of dating apps has increased over the last decade, facilitating the ease of meeting a wide range of people online and in person—for example, those from different regions and cultures, or with similar interests including sexual preference sub-cultures (e.g., BDSM, swinging, polyamory). During the 2020 pandemic the UK government implemented two lockdowns which resulted in a restriction of movement. Pre-pandemic, dating apps offered users a variety of options to meet others ranging from texts, voice and video calls to meeting in person for a date, intimacy, and/or sex [3]. The latter three would be severely constrained during the lockdowns of 2020. 

Previous studies show that many people typically seek out companionship, hook-ups, relationships, and intimacy via dating apps [4]. However, what is missing from current research is the dating app experiences of people during the pandemic and of people in mid- and later life. Although much of society is transitioning into living with COVID-19 as an endemic virus [5,6], for some people in society their use of facemasks and shielding are still ongoing. This is especially the case for people who are clinically vulnerable or have life-limiting or life-threatening health conditions [5,7]. 

There is a growing body of work surrounding the pandemic and its impact on people, and communities, from the perspectives of health, wellbeing, technology, and social connections [2,8,9,10]. Technology use intensified during the pandemic across many sectors, industries, and households, enabling necessary tasks to be undertaken such as online grocery shopping, attending church services [11], connecting friends and family via social media platforms [10], or managing their health, and wellbeing [12]. In addition, social distancing and the government’s stay-at-home messaging significantly increased the levels of both social isolation and loneliness for people of all ages [13,14,15]. Furthermore, the different lockdown orders impacted dating apps users who used them specifically to find long-term companionship or an intimate relationship or to engage in sexual activity such as ‘hook-ups’ or to seek out ‘friends with benefits’.

Therefore, the aims of this study included gathering insights and understanding of the pandemic lockdowns on people’s use of dating apps and the impact dating apps had on their health and wellbeing, intimate relationships, and sexual activity. In this paper we present qualitative data from eight online interviews in conjunction with descriptive data from an online survey alongside textual responses to open survey questions from 138 respondents. The findings illustrate how the participants and respondents were using dating apps during the 2020 UK lockdowns. This research contributes to the fields of gerontechnology, gerontology, health, ‘sex tech’, and social sciences. The inter- and multi-disciplinary approach of this work intersects with (sexual) health, wellbeing, and intimacy via the use of dating apps.

### Background Literature

Dating apps have been accessible since 2006 following the creation of the first dating app—MeetMoi [16], followed by Grindr (2009) [17] and Scruff (2010) [18], while Tinder and Hinge have been around for a decade (2012) [19,20]. Nikiforova [21] notes 27% of adults aged between 18 and 24 years and who are users of dating apps have reported feeling lonely [22,23]. Dating apps offer users access to a wide range of intimacy, relationships, one-night stands/hook-ups, sexual preferences, and sub-culture sexual interests [4,10,23,24,25,26]. 

In 2020, 32% of men and 28% of women in America were reported to use dating apps/sites, with most users aged between 18 and 29 years (48%) and usage being lowest in those aged 65+ years (13%). The numbers of non-heterosexual users—55% of lesbian, gay or bisexual (LGB) people—far outweighed the 28% people who identified as heterosexual [23]. From a UK perspective, the majority of dating app users (31.4%) are aged between 25 and 34 years, followed by those aged between 35 and 44 years (25.7%), 18 and 24 years (20.0%), with the least number of adult users (11.4%) aged between 45 and 54- and between 55 and 64-years [3]. Dating app use in the UK is primarily conducted by men (57.1%), with only 42.9% of women using dating apps. The majority of UK dating app users (40.6%) were categorized as low income, followed by 37.5% and 21.9% of people reporting to have high and medium incomes, respectively [3].

Research exploring dating apps shows a growing and wider area of interests and characteristics including loneliness [25,26,27,28], associated risks (e.g., scamming, sexual violence) [29,30,31,32]), infidelity [33,34,35], wellbeing [36], and motivations [37,38,39,40]. Yet, the majority of this work encompassing dating apps focused on younger users rather than adults in mid- and later life. Nonetheless, scholars in the social sciences have explored sex and intimacy in later life (50+ years) including issues such as erectile dysfunction, medication, and/or reduced libido [41,42,43,44,45,46,47,48]. 

Loneliness is a key factor impacting the wellbeing of both older and younger people [49]. Nevertheless, there is limited understanding of the impact of loneliness on those in mid- and later-life [50]. Pikhartova, Bowling, and Victor [51] note how loneliness can be defined as ‘discrepancy between one’s desired and achieved levels of social interaction’ [52]. Furthermore, scholars note the differences between loneliness and social isolation [53], where loneliness is a negative subjective feeling experienced by individuals and social isolation refers to not having connections with other people [54]. Gorczynski and Fasoli [55] note how 7% (~30 million) of Europeans feel lonely. The European Social Survey found there was a greater feeling of loneliness experienced by those people with poor health, who live alone, who are widowed, who earn a low income or who were unemployed, and who either live in Eastern or Southern Europe [56,57,58].

A qualitative study conducted by Jiménez, Conde-Caballero, and Juárez [59] aimed to understand how technology can facilitate greater social interactions and connections with older adults living in the border regions of Spain and Portugal. The conclusions from this study ascertained how older participants rejected the notion of technology, and many of the participants had limited digital skills and competence.

## 2. Methods

### 2.1. Aims and Objectives

The goal of the study was to examine the dating app activity of users aged 18 years and over in the UK during the COVID-19 pandemic. The aim was to understand the relationship between dating app usage and health, wellbeing, sex, and intimacy. The qualitative data presented are based on an online survey conducted between December 2020 and May 2021 and semi-structured interviews conducted between February and April 2021.

### 2.2. Ethical Approval

Ethical approval was granted by the lead institution (The Open University) [HREC/3441/Marston] and subsequently was submitted for secondary approval by the partner institution—Swansea University.

### 2.3. Participant Recruitment

#### 2.3.1. Survey Participants

A total of 138 respondents completed the survey. The characteristics of the survey respondents are given in Table 1. We acknowledge that this is a small sample and does not fulfil the criteria for quantitative significance. Consequently, only the descriptive and qualitative data from the open survey questions are presented here. 

Descriptive data (Table 2, Table 3 and Table 4) shows a change in dating app use by users during the first and second UK lockdowns. 

#### 2.3.2. Interviewees

Participant recruitment for qualitative data collection employed different approaches, including emails to existing mailing lists, social media profiles (e.g., Twitter and Facebook), and utilized purposive and snowball sampling strategies. A total of eight participants were recruited for online, face-to-face interviews, and these included three females and five males aged between 40–54 years. One interviewee self-identified as a trans woman in a polyamorous relationship, one interviewee identified as a gay married man, and one interviewee identified as a lesbian. The remaining sample consisted of four single, heterosexual men and one single heterosexual woman. All interviewees lived in England, UK, and were regular users of dating apps pre-pandemic. All had continued to use dating apps during the pandemic.

### 2.4. Data Collection and Analysis

#### 2.4.1. Survey

The online survey comprised of 64 items across four sections. Some of the survey sections comprised of existing validated surveys [60,61,62,63], developed to measure dating app use, in addition to survey questions being used based on previous iterations of the survey [64,65,66,67,68,69,70,71]. A link to the online survey was included in all social media posts and emails to mailing lists. Respondents’ anonymity was built into the survey which was hosted via Qualtrics. Data analysis was conducted using SPSS version 27 (IBM Corp, Armonk, NY, USA).

Given the small sample size (*n* = 138), we are unable to present meaningful and significant quantitative findings. Therefore, we present the descriptions pertaining to dating app use and reasons for uninstalling and changing dating apps during the pandemic (Table 2, Table 3 and Table 4). Consequently, we are specifically focusing on the open-ended question presented in the online survey:

‘Please describe your experience of using dating apps during the 1st COVID-19 lockdown and the 2nd COVID-19 lockdown’.

- Can you describe your reason(s) for breaking or for considering breaking the rules during the two UK different lockdown periods.
- Can you describe why you did not break UK lockdown rules during these periods.
- Is there anything else you would like to describe about your experiences during the pandemic/lockdown?

The online qualitative data provide an insight into how dating apps were utilized during this period across the UK.

#### 2.4.2. Procedures—Interviews

Online interviews were conducted by three members of the research team (two women and one man) between February and April 2021. The qualitative data were generated through online, one-to-one, semi-structured video interviews, following a semi-structured protocol (see Supplementary file) created by the research team. The semi-structured interview schedule was flexible to allow interviewees’ stories and experiences to organically unfold. 

The project investigator (PI) was the point of contact (POC) for the study and all prospective interviewees emailed the POC. At this, point key interviewee details were recorded such as gender, region of the UK, preference of interviewer (male or female), preference of date/times, type of data apps used, and an agreed pseudonym. Having met the study criteria, the next stage involved the PI introducing the interviewer and interviewee via Zoom, and then they exited the interview. All interviews were audio-recorded and once completed, were automatically saved to a secure folder. The PI downloaded and securely transferred the file to the external transcription company. The external transcription company transcribed each interview verbatim and returned them via email to the PI. 

#### 2.4.3. Qualitative Analysis

Employing Interpretative Phenomenological Analysis (IPA) facilitated the research team to acquire greater insight and knowledge of the interviewee’s experiences of dating apps during the pandemic. There are six stages to IPA data analysis [72]:

- Reading each transcript several times.
- Notation of important descriptive, linguistic, and conceptual qualities.
- Development of emerging themes.
- Condense emerging themes to form super-ordinate (main) themes including sub-themes.
- Consideration of convergences and divergences.
- Main (super-ordinate) themes formed.

IPA is an idiographic approach (a method that focuses on the human and unique experience of the individual) which requires the in-depth analysis of each participant’s data [73].

Each transcription was uploaded to NVivo version 12 by the PI. Each team member independently coded the transcripts following the first four stages of IPA. During the process of analysis, the team met regularly to develop the emerging themes and agree to a consensus of the main (super-ordinate) themes. 

## 3. Results

We are presenting two types of data: data from the open-ended online survey questions and from the one-to-one interviewees. Quotes from the online survey are identified by the respondent number. Quotes from the individual interviews are characterised by the pseudonym of the interviewee, their age, and the region in England. 

### 3.1. Qualitative Findings

#### 3.1.1. Morality, Health, and Law Breaking and COVID-19

Some of the qualitative survey respondents and face-to-face interviewees reported either having broken or considered breaking the law to meet up with someone. Others perceived that a perspective suitor had shown their poor judgement because they had suggested ‘*stretching the rules*’. This was seen as a ‘*betrayal*’ or a ‘*difference in our values*’. Some felt that, at the time, their circumstances of being single and alone justified breaking the rules:

*During COVID 1st lockdown I met inside with someone when we were not allowed because both of us were single and lived alone and were not at that time mixing with anyone else so both felt we were low risk to have it and if we got it would be low risk to spread it further. It was also at that time of very low prevalence in <Southwest of England>.*

[P12]

The quote above is important because it demonstrates the mindset of two people who had met via an app, who were living alone and single, and they believed to be low-risk because they had not been mingling with other people, coupled with the low prevalence of COVID-19 in their local area; this resulted in these two people making a decision to break the law after weighing the risks to each other. 

One person noted their mindset toward meeting up with people, because of the ‘*lack of opportunity*,’ as well as acknowledging that ‘*the risk was too severe*,’ but also they noted their thinking towards users who were interested in meeting up and breaking the law, ‘*if they were that cavalier with meeting me they may have been the same with others, increasing my chances of exposure*’ [P8]. This notion of a ‘cavalier’ approach, while this respondent may have considered breaking the rules given the opportunity, left them cautious of the fact their health could be put at risk by the other person who may have met many other people and been exposed to the Coronavirus. 

Breaking the law was incomprehensible for some survey respondents, and the potential of meeting up with someone was viewed by one respondent as ‘*potentially killing someone*’ [P6], while another respondent noted the fear or risk of ‘*meeting a stranger and potentially catching the virus or getting caught meeting someone*’ [P54]. This insight highlighted a double jeopardy—fear of catching the virus and fear of been caught. The latter resulting in possible punishment (e.g., a fine).

Some app users perceived those engaged in ‘*rule breaking*’ as not worthy ‘*because […] the people I chatted with—they wanted sex and nothing else—I am looking for a relationship*’ [P55]. Yet, breaking the rules may have occurred when users were ‘*keen enough*’ [P56], while one respondent notes ‘*during the second lockdown there was more of this, and many people were trying to meet while the lockdown was in force*’ [P6]. Breaking the rules set out by the government shows that, for many people, their mindset was not necessarily about keeping safe or keeping those around them safe, but instead, it was their emotional or sexual needs that superseded their rationale for rule breaking, with little consideration for the health implications of exposure to the Coronavirus and spreading it. 

The physical restriction imposed by the regulations meant that alternative approaches toward engagement with others were utilized. Some app users accessed sex tech [74] in order to conduct alternative ways of engagement—‘*I would facetime people—sometimes they just wanted to engage in some sort of sexual activities on camera though*’ [P6]. While some virtual engagements were not positive, other respondents had a different outcome. [P35] reported that their experiences of dating apps and virtual dating led to a relationship: ‘*once the rules relaxed in June, each got COVID tested in order to meet up. Met up. Now dating*’. The virtual dates during lockdown facilitated an alternative approach to dating, and because they were using sex tech to connect, while following the rules, this facilitated the two people to decide whether to commit or not in the future. 

Pandemic directives were instilled to protect people. Nonetheless, for some people the notion(s) of rule breaking were worth the effort. Yet, for others conducting alternative approaches to dating in person, the risks were deemed more appropriate and safer. Diane [50 years, London] describes how she broke the law ‘*a couple of times*’, and her reasoning and thought process was ‘*because I think you just crave human contact, intimacy.*’ Diane acknowledged breaking the law to hug a friend; she noted, ‘*I did break the rules a couple of times. But I was really law abiding 90% of the time*’. Diane describes how she ‘*didn’t feel guilty*’ rather, she accounts that *’I was actually glad I did it to be honest, even though things didn’t work out. I just thought actually at least I had a bit of fun*’. Diane shows no remorse for her rule breaking because, for her, physical and emotional intimacy superseded the regulations. Although she acknowledges that she is a law-abiding person for the majority of the time, on this occasion her needs were more important. 

Harry, a 40-year-old man from the Southeast of England describes how he ‘*kept to the rules*’ but also notes how he ‘*would have been OK with seeing people, and it would have been nice to have a support bubble*’. Upon further questioning, Harry describes what if a television celebrity appeared at his door, requesting to come in he ‘*probably wouldn’t have said no*’. Yet, Harry continues noting how ‘*intention and reality are two different things. So, I just don’t know what I’d have done if I’d had the opportunity*’. Conversely, Noah, a 40-year-old man from the East of England, describes how rule breaking was not a consideration for him. He explained, ‘*if they want to break the rules, that’s down to them*’ because ‘*at the end of the day my goal with this lockdown is to get to the end of it so I can see my children on a regular basis*’. Breaking the rules for Noah was not a priority. For example, he chose not to be with his children ‘*because children can’t keep to that two-metre distance. My boy doesn’t even know what two metres is, not even my daughter doesn’t even know what two metres is. So, it was best just to keep to the rules*’. This insight is important because it is an altruistic element to observing the lockdown regulations. Furthermore, Noah describes the impact of this self-imposed isolation that he chose for himself and his children—‘*It would have been too hard*’ because he would not be able to hug them. 

Racheal, a 44-year-old woman from the Southeast, describes how she considered breaking the rules with her ‘friend with benefits’ whom she usually sees ‘*once every two to three weeks*’ and ‘*That feels, if I can see other friends outside, it feels worth having that as my bubble essentially*’. This decision by Racheal was more of a social responsibility and worked within the respective government directives to see each other. 

Support bubbles, whether it was with a friend with benefits or others, were important for Brett (54 years old, London). He also broke the rules. He was in a relationship with a woman, categorized as vulnerable. Brett described how their relationship started prior to lockdown, but their sexual intimacy did not occur until the lockdown measures were rolled out:

*[…] we’ve started sleeping together and have made each other each other’s bubble if that makes sense, and I know that we are the only person other than her daughter that we spend time with indoors. But yes, there have definitely been a couple of nights when either I’ve been at her house, or she’s been at mine when you are not supposed to stay overnight in somebody else’s house.*

Brett and his partner utilized the notion of ‘support bubbles’ as a means of spending time together, while also ensuring their extended engagement with other people was limited within the household numbers. For Racheal and Brett, they chose to replace support bubbles consisting of friends for their lovers or partners. Their actions demonstrate the balancing of personal needs against societal strictures. Indeed, ambiguity and misunderstanding, based on the directives changing weekly or monthly, led Brett to describe how ‘*lots of things fall down between the cracks*’. The uncertainty generated by government ambiguity led to further questioning of the policy, ‘*[…] if I were to decide to go for a really long walk tomorrow and somebody gave me a ticket for being 10 miles from home, you know, I’d fight it, because I would be walking for exercise not for going to see somebody or whatever*’.

The point highlighted above by Brett shows both how people could misunderstand what was expected of them and/or use the system/the laws to their own advantage. However, Brett stated that if he were to receive a fine for something that he believed was legitimate, he would feel compelled to act: ‘*if I saw a rave going on, I’d call the police about it if that makes sense*’. 

During the pandemic and across the UK, each devolved region had different directives, set out by their respective governments. In England, during the time this study was conducted, different areas/counties had different regulations, and crossing a county line could have resulted in breaking the law. Brett reiterates how he does not see himself as a person who would report other people for breaking the law by noting:

*Again, I’m not a curtain twitcher but, you know, actually the sooner people stop behaving like idiots, hopefully the sooner we can start to, you know, find some kind of normal.*

The participants’ and respondents’ narratives demonstrate the confused messaging by national and local government(s), while also expecting people to be responsible in their own decision-making. Tracey, a 52-year-old woman from the Northeast, describes how she ‘*technically*’ broke the law, and had it not been for her daughter living with her, she could have said ‘*this chap was in my bubble*’. Similar to Racheal and Brett, the rationale of a support bubble was used as a justification for being physically intimate with someone else. Previously, Tracey and this person would meet ‘*for walks, and it was raining one day, and I said, “Oh you can come in but sit over there” kind of thing and then yeah, one thing led to another*’. Reflecting on this occasion, Tracey believed there were no risks posed because she had conducted a ‘*risk assessment*’ of the environment (her home): 

‘[I] *just thought he’s working from home, he’s not seeing anyone, I’m at home, I’m really careful at work and most of the time I’m working from …. So, I did a risk assessment. Yes, I did break the rules I suppose*’. This notion of a risk assessment is used as a validation for her actions and decision in including her male friend in her support bubble: ‘*you know I can be really honest about this, but I haven’t broken, it’s technically I suppose breaking*’. However, Tracey acknowledges that her choices and behaviour have broken the COVID-19 restrictions. She goes on to note how she usually has a set of rules in place before she engages in sexual activity:

*I have a three-week rule or a five-week rule. I wouldn’t sleep with anybody till then but actually that gives them that window of opportunity to go to the sexual health clinic and then show me a text to say that they’ve had their test. And I do the same… I’ve always been really, really careful, but I didn’t this time. I didn’t. And that’s not like me at all.*

Although Tracey declares how she is very careful about her sexual health, she believes ‘*the COVID risk I felt was worse than the sexual health screening. There was a COVID risk, and then there was an STI risk*’. This is an interesting insight into Tracey’s behaviour, and, while she generally takes a cautious approach to her sexual health, she acknowledges feeling ‘*really pressurised*’ during the lockdown to relax her typical sexual mores.

The narratives of the participants highlight how emotional and sexual intimacy could take precedence over COVID-19 restrictions. Furthermore, the concept of a support bubble was used as a means of rationalizing sexual engagement/intimacy The respondents to the online survey perceived the rules were set in place for a reason and should not be broken for their own health risks and that of the greater society. 

#### 3.1.2. Self-Surveillance and Moral Signalling

Survey respondents described their motivations for not breaking the rules, but, in some instances, they instead displayed behaviours pertaining to self-surveillance and moral signalling:

*I don’t think I would break the rules like that to meet someone I hadn’t met before, particularly because you don’t know how much you can trust them and how much they’ve followed the rules.*

[P16]

The quote above is interesting because the respondent notes how they would consider breaking the rules with someone who they had met before in a pre-pandemic society, but not necessarily a stranger. Yet, they continue to describe how they ‘*did meet up with someone at one point, but it wasn’t breaking the rules to meet up. Although, I did then break the rules and kiss them—so maybe if I had liked someone enough, I might have met up and broken the rules.*’ [P16]. This insight is using the premise of social bubbles again to be able to meet up with someone whom they may have met on a dating app and also implying that had there been a greater attraction, further rule breaking would have occurred. 

Being alone or separated from someone led one person to describe how they broke the rules during the first lockdown because of how they were feeling after ‘*eight weeks of lockdown’*, [P20] and they ‘*mutually decided that as we were both living on our own’, so* the risk was potentially not severe. Yet, we can see from this respondent, there was a conscious decision to break the rules because their feeling of loneliness superseded the national directives of not mixing. Additionally, this insight shows the impact that government directives and lockdowns placed on people, whether it was the need for sexual and physical intimacy or just the company of someone to whom you are attracted and exploring whether it is going to go somewhere. 

Moral signalling was portrayed by online survey responses, coupled in the rise of reported daily deaths through the media outlets, as heightening respondents’ reluctance to engage with people that they were talking to on dating apps. Respondents describe how daily mortality rates associated to the directives is evident: ’*[…] they are important for both the people to follow looking at mortality*’ [P4] and ‘*Because potentially killing someone in any way is not really part of my personality type!*’ [P6]. While for one respondent, seeing their children in the future was important to them: ‘*I wanted the lockdowns to be over and life back to normal so I can see my kids.*’ [P5].

The quotes above relating to moral signalling show how respondents were seeing the bigger picture of lockdowns, and the unfolding consequences of the Coronavirus were more important than having an elicit meeting with someone to whom they had little attraction or had just started talking to on a dating app. 

#### 3.1.3. Loneliness and Social Isolation

This theme intersects across mental health, lack of physical and sexual intimacy, and the living environment. There is little understanding of the impact on people who were living on their own during the pandemic. 

One respondent describes how their routine of childcare did not take away the feeling of loneliness and the desire for companionship:

*It’s been lonely. So, so lonely. I have my children with me half the time, but when they go back to their mum’s house, I have nothing at all to do and now, nobody to see. I just sit on my sofa watching tv, playing games, and listening to music, trying to stay connected through social media.*

[P3]

For this person, the void of their children leaving enhanced their feelings of loneliness and lack of purpose during these specific moments. Continuing support bubbles also exacerbated the feeling of loneliness for [P3] because ‘*All of my friends are in their own support bubbles; all of my family too. I have nobody and have had to watch as friends and family have gone on and on about how difficult it is and how lonely they are whilst having spouses to lean on*’. 

Support bubbles set out by the government seemed to be, for this person, a hinderance because they did not have anyone to be with and to share the emotional turmoil and to alleviate their feeling of loneliness. Moreover, it could be argued that ‘*Even having kids’*, for this person, did not help because ‘*I’m the adult—having a grownup around makes such a difference. Plus, they go to bed, and I’m back on the sofa*,’ [P3]. Yet, what this person was craving was adult company, conversation, and even sexual/physical intimacy because, as they note, once their children are in bed, they are alone again, and they perceive the impact of the pandemic as a year taken away from them, by not being able to date and meet new people because ‘*I’ve missed out on a year of dating, and, thanks to the vaccine being so slow […] I’ll likely miss out on another before I get to go out and meet people again*’. Although there is acknowledgement and positivity around the vaccine, [P3] is reconciling themselves to living in a different society with new social norms because ‘*Even then, it won’t be as it was. I fear that I may have missed the boat when it comes to meeting someone and am trying to readjust my expectations accordingly*’ [P3].

Conversely, one respondent describes how the UK directives did not cater to or consider people who are in polyamorous relationships:

‘*It’s been difficult when the rules don’t cater for, e.g., polyamorous families, and assume nuclear monogamous families. It has been useful as a space to experiment with being more open about gender identity and expression.*’ [P7]. Across the directives, there was no consideration for people who do not lead a conventional life and, instead, choose to have and conduct a polyamorous relationship, which may involve the individuals living in different homes rather than under one roof. This too may have had greater consequences for individuals practicing polyamory because they may have been forced to decide to change their living circumstances, by moving in together or choosing to be alone or choosing one partner over another. 

The survey was deployed across the UK during the second lockdown, and the various directives set out by the devolved government (England, Northern Ireland, Scotland, Wales) varied considerably [2]. At the time of survey deployment, it was unclear how this second lockdown would impact the health and wellbeing of people, and two respondents describe their feelings towards the second lockdown. For example, [P9] states, ‘*It’s as bad as it was before, if not worse*’ [P9], while [P11] notes that they ‘*[…] felt more sad and lonely than I have ever felt before because I find it hard to admit to friends and family how sad and lonely I am*’. Although, for these people, the apprehension and fear of another lockdown impacts their feeling of loneliness, and, for [P11], they describe how they cannot be truthful to their friends or family; another person describes how lockdown has been a learning process for them: ‘*Both lonely but a lesson in liking my own company. Lack of physical contact has been the hardest*’ [P17]. While [P9 and P11] have not described the lack of physical or sexual contact as the driver for their feelings of loneliness, [P17] does acknowledge this, and, for many people, we can assume that it is the implementation of lockdowns—taking away any opportunities of physical/sexual contact that impacts the most, and it could be described as the invisible barrier within our society at that moment in time. 

Interviewees described the impact of lockdown relating to their feelings of loneliness and social isolation but also were seeking alternative ways to continue meeting people when permitted with the lifting of restrictions. For example, Racheal reported that during ‘*mid-lockdown where I was probably—oh, I did few speed dating events. So there probably was a time mid-pandemic where I was sort of quite feverous in my approach to it*’. Racheal notes how ‘*it’s very unusual for a person to spend that much time on their own*’, and she was ‘*just seeing if there was anyone out there*’ by attending some dating events. Racheal acknowledges her loneliness, and she continues to describe how the occurrence of a second lockdown being implemented ‘*without having pinned someone down to at least something*’ was something she did not want to experience again, having experienced the first lockdown ‘*entirely on my own*’. Yet, building a connection on a dating app can be difficult, and Racheal notes how dating app users were not ‘*seriously looking*’ for dates because of the impending winter lockdown. 

The goal of using apps is to find a relationship, but instead, Racheal describes how the character flaws of a person can be overlooked; instead of building the foundations and seeking out a positive connection, there are instead ulterior motives as Racheal describes:

*[…] you end up chasing dates or people for the wrong reasons. You try and nail something down for the sake of a potential relationship as opposed to actually: oh you’re interesting, we’re connecting in the right way, let’s pursue that. And you sort of end up chasing.*

This insight into dating apps and user behaviour may have been exacerbated during the pandemic because individuals did not want to experience the feeling of loneliness and social isolation again. Therefore, chasing a person or settling for second best was (for some people/users) better than nothing, better than being alone. 

Noah discussed how he categorises friendships, and, although he may have cyberfriends, in his opinion ‘*a* [real] *friend is go to the pub, go shopping, go to the cinema. That’s what I class as a friend. You do social activities*’. During the pandemic, Noah describes himself as a ‘*lonely person*’ who keeps to himself and does not ‘*want to burden*’ others. However, although he experienced intense loneliness in lockdown, it also afforded him the opportunity for growth; he says: ‘*I learnt so much about myself being in lockdown, which I never thought possible*’.

This section has presented insights into the feelings of loneliness and isolation experienced in lockdown and the need for adult company. In some instances, this meant participants settled for ‘second best’ when it came to seeking out a partner. For others, it was an opportunity for growth and self-development. 

## 4. Discussion

This paper primarily focuses on and presents two types of qualitative findings: 1. qualitative findings from an online survey deployed between December 2020 and May 2021 and 2. qualitative data collected via online, one-to-one interviews conducted between February and April 2021. Data analysis identified three key themes across the narratives: 1. Morality, health, and law breaking and COVID-19, 2. Self-surveillance and moral signalling, and 3. Loneliness and social isolation. 

The analysis explored how the participants perceived the use of dating apps to moderate their existential emotions and behaviours experienced during the pandemic. Although the participants described their behaviour(s) and communication with app users, overall, the notion and execution of conducting physical contact was limited. Narratives indicated that consideration had been given by some participants to break the law by meeting up with someone. For others, this action was viewed negatively because they perceived this type of behaviour as not being a good citizen as well as risking the possibility of extending lockdowns. It was evident that using apps was perceived as a mode of engaging and communicating with people to relieve boredom and alleviate the feeling of loneliness. At the heart of either consciously breaking the law or even considering it was the feeling of loneliness and the need for physical contact—whether it is a hug or sexual intimacy—and demonstrates the importance of social relationships to wellbeing. However, the findings of this study highlight how many of the participants were not necessarily using dating apps during this period to engage with sexual activity but more for companionship and online communication because they were feeling lonely and isolated. The current literature surrounding dating apps, including [4,21,22,23,24,25,26,31,32,33,34,35,36,37,38,39], aims to understand various characteristics, and, indeed, this work aligns and contributes to the existing scholarly work in the disciplines of sex research, gerontology, health and wellbeing, and social sciences. This work contributes to the fields of social sciences, gerontology, human computer interaction because of the inter- and multi-disciplinary nature of this study, coupled with the qualitative findings (via online, one-to-one interviews), primarily focusing on adults aged 40–55 years. To date, there is a paucity of published work in this area focusing on middle aged and older adults and their dating app use. 

However, the narratives around rule breaking show how individuals attempted to justify and rationalize their behaviour. Further, it was noted in the survey responses that different self-imposed rules that were being applied depended on the circumstance—for example, if there was a rave going on, one participant noted they would have informed the police while smaller groups were seen in a more accepting light. 

Moreover, we speculate how loneliness was highlighted as a key theme in this work, and we provide narratives from interviewees and survey respondents alike who describe their sense of loneliness and of feeling lonely during this period. For many people they chose not to break the rules even though they may have been feeling lonely because they wanted the pandemic to end, and, with the cases of COVID-19 increasing, they probably felt that it was never going to end. The interconnections associated to the themes identified are connected to government directives that were reducing freedom of movement and change in social interactions, work, and our pre-pandemic routines. 

We have shown how dating apps during this period were used for different reasons, primarily enabling users to connect with people. Previous habits conducted via apps were not possible, as Stuart who, with his husband, used Grindr to invite a third person into their marriage for a ‘hook-up’ describes. Although some users were using apps as a way of continuing to find their ideal partner, respondents noted how they used the apps as a way of reliving boredom, and, as noted, there was a lack of enthusiasm because of limitations placed on society which limited freedom. Finally, dating app use did change, as shown in our findings (Table 2, Table 3 and Table 4), and this is supported by the qualitative findings presented, whereby many of the quotes presented signalled greater morality, the (un)consciousness of rule breaking, and the experience of loneliness by users which, in turn, led to a change in dating app behaviour and use during these two periods. 

### Strengths and Limitations

A strength of this work is that this is the first piece of work, to the best of the authors knowledge, to be conducted on user’s experiences of dating app use during COVID-19. There is an increasingly strong scholarly interest in dating apps and their usage. However, it is mainly focused on the changes made within apps at the start of the pandemic [72] or taking an exploration through a student lens [73] or the incentives and business model of Tinder [74]. Although the American Association of Retired Persons (AARP) has provided information for older users interested in using dating apps or dating websites [75], there still remains a gap in the literature and in the field of gerontology surrounding dating apps, and sex tech use by adults in mid- and later life. Moreover, existing scholarly articles [21,22,23,24,25,26,27,28,29,30,31,32,33,34,35,36,37,38,39] have primarily focused on younger users of dating apps, with the exception of Marston and colleagues [4] who conducted a review of existing dating apps and how this type of sex tech could be utilized by older adults and people with life-limiting and life-shorting conditions. 

However, the qualitative data shows how loneliness and social and sexual intimacy were drivers for using dating apps during this period. This has implications for the future from a public health perspective, especially how digital transformation was exacerbated throughout society, and the authors believe this work can feed into future public health narratives surrounding interventions to combat loneliness and social isolation. The interviewees are categorized as Generation X [76], and with this, many existing initiatives are targeting adults aged 60+ years. Therefore, if people are presenting loneliness and social isolation in mid-life, there is the possibility that existing interventions and initiatives may not be successful. 

This study presents insights from users categorized as Gen X [76], who are rarely considered when investigations are exploring dating apps or technology in general [6,14,77,78,79]. Therefore, this work is unique from the standpoint of gerontology, sex tech, and social sciences. In this manuscript we present qualitative findings from online interviews pertaining to discourse through the lens of Gen X’ers who will be the next ageing cohort in our society to follow the Baby Boomers, and both cohorts have very different experiences and understandings surrounding the use of sex tech and technology in general. Yet, as we look to the future, scholars, industry professionals (e.g., sex tech), and policy makers need to start to understand the differences between different cohorts because, if not, when Gen X reach later life, there will not be the appropriate initiatives in place to combat social issues. 

A significant constraint to the findings of this project is the small sample size of the online survey. Consequently, the statistical analysis of the relationships and associations between loneliness, health, wellbeing and dating app use was limited. Nonetheless, we have presented some descriptions pertaining to specific dating app use during the pandemic, coupled with the qualitative data from the online interviews to show how Generation X uses dating apps. However, one solution that could have been considered was to keep the online survey open for longer (past May 2021) although that was not possible for practical reasons. The research team utilized existing networks and social media specifically for the online survey which included Age NI. However, greater onboarding of national organisations such as Age UK and other franchises such as Age Cymru, the Campaign to End Loneliness, in conjunction with grass-root networks, may have provided greater survey responses to allow a larger sample size. 

Regarding the interviewees, they were all recruited from different regions of England. Future work should consider and recruit interviewees from across the devolved nations of the UK. 

## 5. Conclusions

The findings presented here have shown three themes derived from qualitative analysis and highlights how dating app use during this time was used for alleviating loneliness and played a role in social, sexual, and physical connections and intimacy. This project was specifically a UK-wide study, and although many of the online survey respondents were located outside of the UK, we believe given the popularity of dating apps, coupled with the importance of loneliness, health, and wellbeing, there is a need to extend this work to afford greater understanding of adults, specifically Generation X, who were the core interviewees and who described their experiences of feeling lonely and loneliness in mid-life. Extending and scaling this work as we transition into a post-pandemic society would afford greater understanding of people’s behaviour in challenging socio-political circumstances. For example, this study captured people’s responses to specific and diverse government directives while tackling COVID-19. 

Future investigations are needed to understand the use and motivations of dating apps by people in mid- to later life because, from a gerontological standpoint, people who are categorized as Generation X are going to be the next ageing cohort. To date, the field of gerontology and gerontological research does not focus its efforts on this cohort, and, instead, it is packaged and narrated in the way of exploring intergenerational behaviours, employing a life course perspective. Given how the interviewees and most survey respondents were over the age of 40 years, there is a clear need to understand dating app use in mid- and later life.

## Figures and Tables

**Table 1:** Survey Characteristics.

Age Group (Years)	*n* = 138 (%)
18–29	19 (14)
30–39	25 (18)
40–49	40 (29)
50–59	24 (17)
60+	6 (4)
Region of the UK	
England	95 (69)
Scotland	12 (9)
Northern Ireland	6 (4)
Wales	9 (7)
Other	4 (3)
Type of Community	
Rural < 2500	10 (7)
Small town < 2501–10,000	21 (15)
Suburban 10,001–50,000	33 (24.9)
Metropolitan/City 50,001>	50 (36)
Gender	
Male (inc. transgender men)	33 (24)
Female (inc. transgender women)	80 (58)
Prefer to self-describe as (non-binary, gender fluid, agender)	1 (1)
Marital status	
Living with partner	3 (2)
Married	7 (5)
Widowed	1 (1)
Divorced	28 (20)
Separated	6 (4)
Never married	3 (2)
Single	64 (46)
Other	2 (1)
Ethnicity	
White British	92 (67)
White Scottish	1 (1)
White Irish	2 (1)
White other	11 (8)
Mixed White/Asian	2 (1)
Mixed Other	1 (1)
Asian/Asian British Bangladeshi	1 (1)
Asian/Asian British Indian	4 (3)
Other ethnicity	2 (1)
Prefer not to describe	1 (1)
Education	
High School/GCSE/‘O’ Levels	4 (3)
Some college, no degree (e.g., BTEC, NVQ, A-Levels)	16 (12)
Bachelor’s degree (3-yrs)	40 (29)
Bachelor’s degree (4-yrs)	8 (6)
Master’s degree	30 (22)
PhD	11 (8)
Professional degree (e.g., MD)	5 (4)
Disability status	
Yes	14 (10)
No	100 (73)
Self-isolation during the first &second UK lockdowns	
Yes	25 (18)
Sometimes	19 (14)
No	70 (51)

**Table 2:** Dating app use during the pandemic.

	Use of Dating Apps during the First and Second UK Lockdowns *n* = 138	
	1st Lockdown N (%)	2nd Lockdown N (%)
Yes	67 (49)	35 (25)
Sometimes	19 (14)	18 (13)
No	18 (13)	24 (17)
I uninstalled the Dating Apps at the beginning of the lockdown	4 (3)	-
Total	108 (78)	77 (56)
Missing responses	30 (22)	-

**Table 3:** Reasons for not using or uninstalling dating apps during the pandemic.

Reasons	Total *n* = 138 (%)
For my mental health	6 (4)
I did not see the point in using dating apps during the pandemic	7 (5)
I could not be bothered	4 (3)
Because I was not able to meet anyone	5 (4)
I was no longer looking to date	6 (4)

**Table 4:** Choice of dating apps change during the pandemic.

Responses	Total *n* = 138 (%)
Yes	16 (12)
Sometimes	10 (7.2)
No	64 (46)
Total	90 (65)
Missing responses	48 (35)

## Data Availability

We are still conducting analysis from this dataset, and we cannot provide the full dataset at present. Persons interested in accessing the data may contact the corresponding author.

## Supplementary Materials

The following supporting information can be downloaded at: https://www.mdpi.com/article/10.3390/healthcare11060897/s1, Online Survey.
