# Supplementary material for: Shiver Me Tinders and Ring a Ding for a Fling—Sex Tech Use during COVID-19: Findings from a UK Study
Source: Healthcare (Basel). 2023 Mar 20;11(6):897. doi: 10.3390/healthcare11060897 (PMC10048256; doi:10.3390/healthcare11060897)
Supplement: Supplementary file 1 [file healthcare-11-00897-s001.zip › healthcare-2199192-supplementary.pdf]

SUPPLEMENTARY FILE: Online Survey

#	Item	Answer options
1	Which region of the UK do you live?	England<br>Scotland<br>Northern Ireland<br>Wales<br>Other (please state)
2a/b/c/d	Please select your County	Each county is listed depending on the answer for Q1 (England, Scotland, Northern Ireland or Wales).
3	What type of community do you live in?	Rural<br>Small town<br>Suburban 10,001-50,000 people<br>Metropolitan/City 50,001> people
4	How old are you (in years)?	Person inserts their age
5	What is your highest level of education? - please select **ONE** answer	Less than high school degree<br>High School/ Senior High school (e.g., O Levels, GED, GCSE)<br>Some college but no degree (e.g., BTEC, NVQ, A-Levels)<br>Bachelor's degree (3-year)<br>Bachelor's degree in college (4-year)<br>Master's degree<br>Doctoral degree<br>Professional degree (JD, MD)

6	Which best describes your ethnicity?	White British<br>White Irish<br>White Other<br>Irish, Gypsy or Romany Traveler<br>Mixed - White & Black Caribbean<br>Mixed - White & Black African<br>Mixed - White & Asian<br>Mixed - Any other mixed<br>Asian or Asian British Pakistani<br>Asian or Asian British Bangladeshi<br>Asian or Asian British - Indian<br>Asian or Asian - Any other Asian<br>Black or Black British - Caribbean<br>Black or Black British African<br>Black or Black British - Any other Black<br>Arab<br>Any other ethnic group<br>Prefer not to describe
7	How would you describe your gender?	Male (including transgender men)<br>Female (including transgender women)<br>Prefer to self-describe as (non-binary, gender fluid, agender, please specify)<br>Prefer not to say
8	Do you identify as transgender or have a transgender history?	Yes<br>No<br>Prefer not to say
9	Please select the sexual orientation category that best represents you:	Heterosexual (straight)<br>Gay man<br>Gay Woman / Lesbian<br>Bisexual<br>Non-binary<br>Pansexual<br>Prefer not to say<br>Prefer to self-describe

10	Marital Status	Single<br>Living with partner<br>Married<br>Widowed<br>Divorced<br>Separated<br>Never Married<br>Civil Partnership<br>Prefer not to say
11	How many people are living or staying at your address?	1<br>2<br>3<br>4<br>5<br>6<br>More than 6
12	Which statement best describes your current employment status?	Furloughed<br>Working (paid employee)<br>Working (self-employed)<br>Not working (temporary layoff from a job)<br>Not working (looking for work)<br>Not working (retired)<br>Not working (disabled)<br>Not working (other)<br>Prefer not to answer<br>Carer<br>Other

13	Which of the following industries most closely matches the one in which you are employed?	Education (e.g., Teacher/ Academic/Researcher/Doctoral Student)<br>Entertainment/Tech Industry<br>I am a student (e.g., part time, full time)<br>Forestry, fishing, hunting or agriculture support<br>Real estate or rental and leasing<br>Mining<br>Professional, scientific or technical services<br>Utilities<br>Management of companies or enterprises<br>Construction<br>Admin, support, waste management or remediation services<br>Manufacturing<br>Educational services<br>Wholesale trade<br>Health care or social assistance<br>Retail trade<br>Arts, entertainment or recreation<br>Transportation or warehousing<br>Accommodation or food services<br>Information<br>Other services (except public administration)<br>Finance or insurance<br>Other
14	Do you consider yourself to have a disability?	Yes<br>No<br>Prefer not to say
15	Have you had to self-isolate during the **1st Covid-19 lockdown and the 2nd** Covid-19 lockdown?	Yes<br>Sometimes<br>No
16	If you answered **YES or SOMETIMES**- please describe why?	Respondent writes their answer
17	If you answered **No** - please describe why?	Respondent writes their answer

18	During the 1st Covid-19 lockdown **and the** 2nd Covid-19 lockdown are you, or did you use Dating apps?	Yes<br>Sometimes<br>No<br>I uninstalled the Dating Apps at the beginning of lockdown
19	Why did you choose not to use or uninstall the dating apps at the beginning of the 1st Covid-19 lockdown and the 2nd Covid-19 lockdown? (select all that apply)	For my mental health<br>I did not see the point in using Dating Apps during the pandemic<br>I could not be bothered<br>Because I was not able to meet anyone<br>I was not longer looking to date
20	Please describe your experience of using dating apps during the 1st Covid-19 lockdown **and the** 2nd **Covid-19 lockdown**	Respondent writes their answer
21	Are you using Dating Apps during the 2nd lockdown period?	Yes<br>Sometimes<br>No
22	Select all dating apps that you are **currently or were** using during the 1st and 2nd Covid-19 lockdowns	I have still not reinstalled any dating apps<br>Badoo<br>Tinder<br>Bumble<br>Plenty of Fish (POF)<br>Grindr<br>Zoosk<br>Lumen<br>eHarmony<br>Match.com<br>Happn<br>Scruff<br>Coffee meets Bagel<br>Other

23	Select all dating apps that you were using or currently using during the 1st Covid-19 lockdown (25.03.2020) and the 2nd Covid-19 lockdown (05.10.2020)	I was not using any dating apps during Covid-19 lockdown<br>Badoo<br>Tinder<br>Bumble<br>Plenty of Fish (POF)<br>Grindr<br>Zoosk<br>Lumen<br>eHarmony<br>Match.com<br>Happn<br>Scruff<br>Coffee meets Bagel<br>Other
24	Did your choice of dating app(s) change during the pandemic?	Yes<br>Sometimes<br>No

25	During the 1st Covid-19 lockdown and the 2nd Covid-19 lockdown - what was your motivation(s) for use dating apps? (Please select **all** that apply).	Friendship<br>Finding love<br>Having a 1-night stand<br>I'm a swinger<br>I'm addicted to sex<br>I'm trying to understand my sexuality<br>I want to find an older woman<br>I want to find an older man<br>I want to find a younger woman<br>I want to find a younger man<br>I have fetishes/kinks<br>I am into BDSM<br>To experience anal<br>To cheat on my partner/spouse/girlfriend/boyfriend<br>I'm interested in group sex<br>Boredom<br>Shibari<br>To build a connection with someone<br>To communicate with someone/people<br>Pegging<br>Because I am lonely<br>To get over my ex<br>Peer pressure, because my friends suggested I use them<br>To connect with other people with the same sexual orientation<br>To get to know people with the same sexual orientation.<br>To meet singles with a similar sexual orientation.<br>To pass the time<br>For fun<br>Because it is entertaining<br>To relax<br>As a break at work or during a study period<br>Other

26	During the **<u>pandemic</u>**, how often did you use these dating app(s).	Never<br>Once a month<br>2-3 times a month<br>Once a week<br>4-5 times a week<br>Daily<br>4-6 times a day<br>Once an hour<br>2 or more times per hour
27	Did your use of dating apps increase during the **<u>pandemic</u> (including lockdown periods)**?	Yes<br>Sometimes<br>No
28	During the <u>**1st** Covid-19 lockdown</u> and the <u>2nd Covid-19 lockdown</u>, did you break the UK Government guidance to meet up with someone?	Yes<br>Maybe<br>No<br>I thought about it, but decided against
29	Can you describe your reason(s) for breaking or for considering breaking the rules during the UK 2 different lockdown periods	Respondent writes their answer
30	Can you describe why you did **NOT** break UK lockdown rules during these periods	Respondent writes their answer
31	Do your friends or family know that you use dating apps?	Yes<br>No<br>Unsure
32	Do you think there are risks to using dating apps?	Yes<br>No<br>Possibly<br>Don't know

33	Select all types of risk that **<u>you</u>** have experienced	Safety risk<br>Sexual health (e.g., contracting STDs)<br>Pregnancy<br>Rape<br>Increased anxiety<br>Bullying/trolling<br>Scamming<br>Sexuality Harassment<br>Poorer mental health<br>Contemplating suicide<br>Sexting<br>Sexual images (receiving images from a contact)<br>Embarrassment<br>Loneliness<br>Catfishing<br>Bread crumbing<br>Ghosting<br>I have not experienced any of the above<br>Other
34	What would put you off from using or deleting a dating app?	Limited dating experience<br>Not tech savvy<br>I lack confidence to put myself out there on a dating app<br>I am concerned about having my personal information online<br>I am concerned about having my photo(s) online<br>I am concerned about what others might think of me<br>I am not looking for a 1-night stand<br>I am a virgin<br>I am fed up experiencing bad behaviour (e.g., in appropriate content)<br>I am looking for companionship<br>Other
35	During the <u>1st Covid-19 lockdown</u> and the <u>2nd Covid-19 lockdown</u> did your Dating app profile change (e.g., type of information shared)	Yes<br>Sometime<br>No

36	You answered **YES** or **SOMETIMES** to changing your profile. Please describe how your profile changed.	Respondent writes their answer
37	Do you share your Dating app experiences with anyone?	Yes<br>Sometimes<br>No
38	Who do you share your experiences with? (Select **ALL** that apply)	My sibling(s)<br>My parents<br>My children<br>My grandchildren<br>Twitter<br>Facebook<br>My friends<br>My work colleagues<br>Other
39	Prior to the 1st Covid-19 lockdown and the 2nd Covid-19 lockdown did you decide to move your partner/boyfriend/girlfriend/FWB into your living space? (Select 1 answer)	Yes, I asked him to move in with me<br>Yes, she asked me to move in with her<br>No, but I thought about asking them<br>Most definitely not<br>I wish I had asked them, I was lonely<br>No, but I wish they had asked me<br>Yes, my FWB moved in<br>No, but my FWB asked me<br>No, I lived alone
40	During the 1st Covid-19 lockdown (25.03.2020) and the 2nd Covid-19 lockdown (05.10.2020) did you have sex primarily because you felt obliged to or that it was your duty?	Almost never/never<br>A few times (much less than half the time)<br>Sometimes (about half the time)<br>Most of the time (much more than half the time)<br>Almost always/always
41	During the 1st Covid-19 lockdown and the 2nd Covid-19 lockdown, did your sexual activity increase with your partner/ girlfriend/ boyfriend/FWB/spouse?	Yes<br>Sometimes<br>No<br>I was on my own

42	During the 1st Covid-19 lockdown and the 2nd Covid-19 lockdown, how often did you, your partner share the same sexual likes/ dislikes?	Almost never/never<br>A few times (much less than half the time)<br>Sometimes (about half the time)<br>Most of the time (much more than half the time)<br>Almost always/always<br>Not applicable
43	At the time of the UK Government restrictions easing, did you living arrangements with your boyfriend/girlfriend/partner/FWB/spouse change?	No, they moved out as soon as it was possible<br>Yes, and we are planning to make more of a commitment<br>Yes, our relationship has grown stronger because of lockdown<br>No, it was a terrible mistake<br>Not applicable
44	Please describe how your living arrangements changed during this period	Respondent writes their answer
45	During the 1st Covid-19 lockdown and the 2nd Covid-19 lockdown, how often did you feel emotionally close to your partner when you had sex together?	Almost never/never<br>A few times (much less than half the time)<br>Sometimes (about half the time)<br>Most of the time (much more than half the time)<br>Almost always/always<br>Not applicable
46	Do you think the use of Dating Apps is the best way to meet new people, potential partners/ spouses?	Yes<br>No<br>Unsure<br>Other
47	Whether or not you have had any recent sexual activity, during the 1st Covid-19 lockdown and the 2nd Covid-19 lockdown, how worried or concerned have you been about your overall sex life?	Not at all worried or concerned<br>A little bit worried or concerned<br>Moderately worried or concerned<br>Very worried or concerned<br>Extremely worried or concerned
48	How important does sex play a part in your life today?	Extremely important<br>Very important<br>Moderately important<br>Slightly important<br>Not at all important

49	How often do you feel that you are “in tune” with the people around you	Never<br>Rarely<br>Occasionally<br>Often<br>Always
50	There is always someone I can talk to about my day-to-day problems<br>I miss having a really close friend.<br>I experience a general sense of emptiness.<br>There are plenty of people I can lean on when I have problems.<br>I miss the pleasure of the company of others.<br>I find my circle of friends and acquaintances too limited.<br>There are many people I can trust completely.<br>There are enough people I feel close to.<br>I miss having people around me.<br>I often feel rejected.<br>I can call on my friends whenever I need them	5-pt Likert for each statement.<br>1=None of the time, 5=All of the time
51	**FAMILY**: Considering the people to whom you are related by birth, marriage, adoption, etc.:<br><br>1. How many relatives do you see or hear from at least once a month?<br>2. How many relatives do you feel at ease with that you can talk about private matters?<br>3. How many relatives do you feel close to such that you could call on them for help?	6-pt Likert for each statement.<br>1=None, 2=One; 3=Two; 4=Three or Four; 5=Five thru Eight; 6=Nine or more
52	**FRIENDSHIPS**: Considering all of your friends including those who live in your neighbourhood:<br><br>1. How many of your friends do you see or hear from at least once a month?<br>2. How many friends do you feel at ease with that you can talk about private matters?	6-pt Likert for each statement.<br>1=None, 2=One; 3=Two; 4=Three or Four; 5=Five thru Eight; 6=Nine or more

	3. How many friends do you feel close to such that you could call on them for help?	
53	Do you have the Internet at home?	Yes<br>No
54	How do you usually access the Internet? Select **ALL** that apply.	via a Smartphone<br>via a PC/Laptop<br>via a Tablet<br>via the TV<br>via multiple devices
55	How frequently do you use the Internet?	More than once a day<br>About once a day<br>More than once a week<br>Every other day<br>More than once a month
56	What do you use the Internet for? Select **ALL** that apply.	Job searching<br>Searching for Health Information<br>Using Facebook<br>Using Twitter<br>Streaming Netflix<br>Streaming BBC iPlayer<br>Streaming other<br>Downloading (e.g., music, TV series etc.)<br>Playing games<br>Listening to music<br>Reading<br>I use the Internet for work<br>Online Banking<br>Emailing<br>Online Shopping<br>Searching for Information (e.g., weather, fact checking etc.)<br>Dating<br>Booking travel arrangements<br>Booking holidays<br>Booking events (e.g., music events)<br>Accessing gambling sites<br>Other
57	Do you use social networking sites (E.g., Facebook, Twitter, Snapchat, Instagram, Pinterest, LinkedIn)?	Yes<br>No

58	Which social media & communication platforms do you use? Select **ALL** that apply	Facebook<br>Twitter<br>Instagram<br>Snapchat<br>LinkedIn<br>What's App<br>Viber<br>Messenger<br>Pinterest<br>Tumblr<br>YouTube<br>Telegram<br>WeChat<br>Tik Tok
59	How long have you been using social networking sites (Facebook, Twitter, Instagram, Pinterest, LinkedIn)?	10-15 years<br>5-10 years<br><5 years<br><1 year
60	How frequently do you use social networking sites?	Daily<br>4-6 times a week<br>2-3 times a week<br>Once a week<br>Never
61	Which communication platforms do you use the most? Select **ALL** that apply.	WhatsApp<br>Face Time<br>Facebook messenger<br>Microsoft Teams<br>Zoom<br>Skype/Business<br>Telegram<br>Viber<br>SMS<br>Telephone call<br>Snapchat<br>Other

62	Why do you use social media sites? Select **ALL** that apply.	To share information<br>To stay connected to friends<br>To stay connected with family<br>To share photographs<br>To organise events<br>To take part/follow events & groups that I am interested in<br>To keep up to date with the latest news<br>To express my opinions and views<br>Other
63	Who introduced you to social networking? Select **ALL** that apply.	Myself<br>Friends<br>Family<br>Spouse/partner<br>Child<br>Grandchild
64	Is there anything else you would like to describe about your experiences during the pandemic/lockdown?	Respondent writes their answer

TOPIC GUIDE: Online Face-to-Face Interviews

A qualitative study about the experiences of older adults using Dating Apps pre-during-and post the Covid-19 pandemic as a way to connect with individuals socially, emotionally, and sexually.

Example interview topic guide for Older Adults using Dating Apps prior to and during the Covid-19 Pandemic.

In keeping with the iterative, inductive approach for the study, this topic guide is a flexible working document that will be adapted at each interview to ensure that interviews are led by participants' narratives. The document will be revised throughout, to allow us to explore emerging themes and gaps identified in early interviews during later data collection. This detailed example version lists main questions and prompts/probes that may be helpful to interviewers, but we will also develop as a shorthand version as we progress with interviews and become more familiar with the guide.

Opening interview

In this study we want to find out about your experiences of using Dating Apps (e.g. Tinder, Bumble, Grindr, Lumen etc.) pre-and-during the Covid-19 pandemic, as a way of **socially, emotionally, and sexually connecting with a person or people**.

- Thank you for talking with me today.
- The interview is likely to last for around 60minutes – although it may go on for longer.
- If there are any questions you do not feel like answering please say and we can move on.
- Can you tell me a little bit about yourself? *(age, living situation, relationship status, sexuality, employment, retired)*

Can you tell me a little bit about your digital technology use?

- What type of digital devices do you use?
- How long have you been using digital devices (e.g. smartphone, Internet, PC etc.)?
- How frequently do you use your digital devices?
- How did you learn to use the different digital devices?
- Did you encounter any difficulties to using your digital devices?
- What were your motivations for using your digital devices?
    - Have your motivations changed for using digital devices/technology?

Can you tell me a bit about your use of social media platforms (e.g. Twitter, Facebook etc.)?

- What type of social media platforms do you use?
- How long have you been using your chosen platforms?
- How frequently do you use your chosen platforms?
- What are your motivations for using your chosen platforms?
- Did you encounter any difficulties to using your chosen platforms?

How are you feeling in yourself at the moment?

- How have you been managing your own social pre-and-during the pandemic?
    - How has your behaviour changed over the last X- number of months in relation to your social wellbeing?
    - How has your behaviour changed over the last X- number of months in relation to your emotional wellbeing?
    - How has your behaviour changed over the last X- number of months in relation to your sexual wellbeing?
    - How has your behaviour changed over the last X-number of months in relation to your social, emotional, and sexual wellbeing?
- How have other people been supporting you emotional and sexual wellbeing?
    - Would you like to comment on the behaviour of others that you may have experienced?
- Can you describe whether your mental wellbeing has been affected by lockdown?
- Are you doing anything different to manage your emotional and/or sexual wellbeing during the pandemic?
- Have you been using any technology to support your own emotional and/or sexual wellbeing?
- Have you considered breaking lockdown rules to meet up with someone?
    - IF YES – what were your motivations for breaking the lockdown rules?
    - IF NO – what stopped you from breaking the lockdown rules?

Can you tell me about your use of Dating Apps?

- Did you have to upgrade your phone because of wanting to use/install Apps?
    - How did you feel about upgrading your phone for this activity?
- What type of Dating Apps do you use?
    - Do you have a preferred app?
    - Can you describe why?
    - Can you describe what your experiences of other Apps has been like?
- How were you introduced to Dating Apps?
- How long have you been using Dating Apps?
    - How frequently do you check your apps?
    - Can you describe your behaviour of using Dating Apps, prior, during and after lockdown?
        - What kind of changes (if any) did you experience?

            - Increase/decrease of checking the App(s)?
            - Increase/decrease of communicating with people?
            - Downloaded more Apps?
- Can you describe whether there were any specific times of the day when you used the Apps?
    - Do you have a preferred app?
    - Can you describe why?
    - Can you describe what your experiences of other Apps has been like?
- What are your motivations for using Apps & creating a profile(s)?
    - To make friends/meet new people.
    - Emotionally, sexually connect with people.
    - Company (sometimes just nice to talk to someone else – not exactly lonely)
    - Loneliness or socially isolated?
    - Specific kinks?
    - To meet people with a similar vibe (e.g., music choices/Instagram links etc.?
- Have you encountered any usability issues while using Apps?
    - Creating an account & including details
        - What kind of details did you include (e.g. hobbies, interests, intent etc.)?
    - Uploading (profile) images
    - Communicating/messaging with others
    - Using app features e.g., swiping
    - Have you linked your dating app to any other social media platforms (e.g. Instagram etc.).
        - If NO – would you ever consider linking your accounts? Yes/no – please describe.
        - If YES – can you describe why you linked your accounts?
- Who knows about your use of Dating Apps?
    - How have these people (friends/family) reacted to you using Dating Apps?
- Can you describe what kind of positive and/or negative experiences you have experienced while using Dating Apps?
    - How did that affect you?
- Can you describe what kind of interaction (e.g. video calls via the app or other platform), communication you have experienced while using Apps?
    - Anything positive? Genuine connections (arranging to meet -virtually or in person/ whether allowed or not?
    - For example, scamming, Catfishing, Fraud, Bread Crumbing Sexting, proposition by escorts, inappropriate messages?
    - What (negative) impact(s) has this type of behaviour had on your mental health?
    - Can you describe what (if any) coping mechanisms for this behaviour on your emotional wellbeing?
    - Have you conducted this type of behaviour to other users on Dating Apps?
- Can you describe how you think your behaviour might change for the better or worse in the future? With respect to apps and their use?

Socially, emotionally, and sexually connecting with people?

- What kind of connections are you specifically looking for?
    - How many connections have you encountered? [possibly breakdown – socially, emotionally and sexually]
- Have you experienced positive social connections while using Apps?
    - Have you experienced positive emotional connections while using Apps?
    - Have you experienced positive sexual connections while using Apps?
- Have you experience negative social, emotional, and sexual connections while using Apps?
    - Have you experience negative emotional while using Apps?
    - Have you experience negative sexual connections while using Apps?
- Have you implemented or considered implementing any forms of resilience to continue using dating apps in the future?

Loneliness, social isolation, mental health and wellbeing?

- Can you describe how you have managed your sense of feeling lonely and been socially isolated? how have you managed this feeling?
- Can you describe how you have managed your experiences (if any) of feeling lonely, been socially isolated – how have you managed this feeling?
    - Can you describe times when you have felt lonely and/or socially isolated?
    - Can you describe your motivations for developing your social engagement?
    - Can you describe what type of activities that you have chosen to do to build/enhance social engagement?
- Has your mental health and wellbeing been affected by (any) sense of loneliness and social isolation?
    - Have you experienced any
- Did you talk to anyone about your loneliness or social isolation?

How do you connect socially with others?

- What sort of social connections do you have usually *(f2f, online, family, etc)*?
- How has Covid-19 impacted on your social connections?
- What differences are there in the way that you connect socially?
- Can you describe if there are any differences in how you have used technology during the pandemic to connect with people?
- How are you managing lockdown and socially isolating?
    - Following guidance?
- How do your experiences of social isolation now compare with your everyday life before the pandemic?

Are you socially isolating or shielding at the moment?

- If you are socially isolating, why and when did you start?

- Have you had to put anything into place to help you isolate or shield? (e.g., support with getting shopping etc.)
- How are you feeling at the moment about having to do this?
    - Was it voluntary/under guidance (e.g., NHS letter)/ to protect someone else?
- What effect do you think this is having on your social, emotional, and sexual relationships?
- What effect do you think this is having on your family/support network(s)?

Closing interview

- How has it been for you talking to me about these issues?
- How do you see the next few weeks/months going, for you?
- Are there any other issues that you would like to discuss that relate to your experiences of using Dating Apps during the pandemic?
- Would you like to hear the results of the study?
- Thank you for participating.
- You will receive your £30 Amazon voucher.
    - Address details will be collected prior to interview. If details are not, then this information can be collected at the end of the interview.
    - I am stopping the recording now.

After the interview we will email the participant the 'Keeping safe and well during the pandemic: Information Sheet' which contains details of who they can contact if they need information, support or are worried about any issue.
